# Enhancement of EPC migration by high-dose lisinopril is superior compared to captopril and ramipril

**DOI:** 10.12688/f1000research.26395.1

**Published:** 2021-01-11

**Authors:** Yudi Her Oktaviono, Hanang Anugrawan Ahmad, Makhyan Jibril Al Farabi, Parama Gandi, Caesar Lagaliggo Givani, Indah Sari Purna Lumeno, Yusuf Azmi

**Affiliations:** 1 Department of Cardiology and Vascular Medicine, Airlangga University, Soetomo Academic and General Hospital, Surabaya, East Java, 60825, Indonesia; 2 Department of Internal Medicine, Airlangga University, Soetomo Academic and General Hospital, Surabaya, East Java, 60825, Indonesia; 3 Postgraduate Degree, Hassanudin University, South Sulawesi, 90245, Indonesia; 4 Faculty of Medicine, Airlangga University, Surabaya, East Java, 60825, Indonesia

**Keywords:** ACE Inhibitors, Coronary artery disease, Endothelial progenitor cells, Migration

## Abstract

**Background: **Angiotensin-converting enzyme (ACE) inhibitors have been shown to promote endothelial progenitor cell (EPC) function. However, the efficacies of different ACE inhibitors in improving the migratory capabilities of ECPs in coronary artery disease (CAD) patients is unclear. This study compared the effectiveness of captopril, lisinopril, and ramipril toward the migration capability of impaired EPCs from CAD patients.

**Methods:** We isolated peripheral blood mononuclear cells (PBMCs), separated EPCs from PBMCs, and divided them into an untreated group (control) and treated groups of captopril, lisinopril, and ramipril at doses of 1mM, 10mM, and 100mM. EPC migration was evaluated using the Boyden chamber assay. Analysis of variance (ANOVA) was performed using SPSS 25.0.

**Results:** This study showed that treatment with captopril, lisinopril, and ramipril starting at the lowest dose (1 mM) increased EPC migration (65,250 ± 6,750 cells; 60,750± 5,030 cells; and 49,500 ± 8,400 cells, respectively) compared to control (43,714 ± 7,216 cells). Increased migration of EPCs was observed by increasing the treatment dose to 10 mM with captopril, lisinopril, and ramipril (90,000 ± 16,837 cells; 79,071 ± 2,043 cells; and 64,285 ± 11,824 cells, respectively). The highest EPC migration was shown for lisinopril 100 mM (150,750 ± 16,380 cells), compared to captopril and ramipril at the same dose (105,750 ± 8112 cells and 86,625 ± 5,845 cells, respectively).

**Conclusions:** Captopril, ramipril, and lisinopril were shown to increase EPC migration in a dose-dependent manner. Low-dose (1 mM) and medium-dose (10 mM) captopril had a larger effect on ECP migration than lisinopril and ramipril. Meanwhile, high-dose lisinopril (100mM) had the highest migration effect, suggesting it may be preferable for promoting EPC migration in CAD patients.

## Introduction

Endothelial dysfunction and impaired endothelial regeneration are thought to play an important role in the pathogenesis of arteriosclerosis in coronary arterial disease (CAD) ^1^. Endothelial regeneration is not only fulfilled by resident endothelial cells but also repaired by endothelial progenitor cells (ECPs) originating from the bone marrow ^2^. ECPs are premature circulating cells, a specific subtype of hematopoietic stem cells that differentiate into endothelial cells *in situ* and promote neovascularization ^3, 4^. Several studies have shown that in patients with CAD, there is a significant decrease in the number and migratory function of circulating EPCs, which leads to impaired neovascularization of ischemic tissue ^5, 6^. Low EPC counts can predict severe endothelial dysfunction, cardiovascular events, and deaths from cardiovascular causes ^7, 8^. It is suggested that intracellular damage and impaired redox balance in EPCs due to oxidative stress are the predisposes of imbalance in vascular pathology ^9, 10^.

Angiotensin-converting enzyme (ACE) inhibitors are widely used in cardiovascular disease and have been shown to be associated with beneficial effects on EPCs in several *in vitro* and clinical studies ^11– 14^. An animal study on mice with increased left ventricular pressure showed that ramipril increases the number and improves EPC migration ^12^. A clinical study in hypertensive patients showed that enalapril and zofenopril reduce EPC levels and prevent vascular damage and carotid intima-media thickening ^13^. In a small clinical trial, administration of ramipril for four weeks in patients with stable CAD augments and increases the functional activity of EPCs, including migration, adhesion, and *in vitro* capacity of vasculogenesis ^15^.

However, no studies have investigated the role of ACE inhibitors of captopril and lisinopril in relation to the EPCs. In addition, the comparison between different types of ACE inhibitors toward the impaired migration function of EPCs remains to be investigated. We aimed to evaluate the effects of captopril, lisinopril, and ramipril on EPCs migration from CAD patients.

## Methods

### Ethical statement

Our study protocol was approved by the Institutional Ethics Committee of Dr. Soetomo General Hospital (945/KEPK/II/2019). Informed consent for peripheral blood sampling procedures and participation in research studies was obtained from all patients before the blood was drawn. We have omitted all data that could reveal the identity of the patients.

### Study population

In the present study, we used peripheral blood samples from the same participants and performed similar methodology to that described in our previous study ^16^. From June 2018 to December 2018, we studied a total of eight patients with stable CAD who underwent coronary angiography. Only patients with the left main coronary artery stenosis of more than 50% or stenosis in other coronary arteries more than 70% were recruited. To prevent the effects of myocardial ischemia on ECP kinetics, we excluded patients with a history of new-onset acute myocardial infarction. In addition, patients with anemia, diabetes, a history of percutaneous coronary intervention, or coronary artery bypass grafting were not included in the study. Physical examination was performed to determine body mass index (BMI) and to assess the vital signs. We also examined the lipid profile and performed echocardiography to assess left ventricular function. The characteristics of the study population are summarized in Table 1.

**Table 1.** The characteristics of the study population.

Variable	Mean ± SD
Age (years)	54.50 ± 4.31
BMI (kg/m2)	25.39 ± 2.13
Heart rate (times/minute)	86 ± 8.68
Systolic blood pressure (mmHg)	137.50 ± 24.35
Diastolic blood pressure (mmHg)	80 ± 7.56
Triglyceride (mg/dl)	97 ± 11.64
LDL (mg/dl)	145 ± 61.11
HDL (mg/dl)	35 ± 7.64
Total cholesterol (mg/dl)	200.50 ± 74.75
Left ventricle ejection fraction (%)	53.5 ± 4.11

BMI, body mass index; HDL, high-density lipoprotein; LDL, low-density lipoprotein; SD, standard deviation.

### Preparation of blood samples and mononuclear cell isolation

We collected 40 ml peripheral blood samples from the median cubital vein following WHO guidelines on drawing blood ^17^. From freshly drawn heparinized blood, we isolated peripheral blood mononuclear cells (PBMCs) using Ficoll Histopaque 1077 (Sigma-Aldrich, USA). Briefly, peripheral blood was diluted 1:1 with phosphate buffer saline (PBS) + 2% fetal bovine serum (FBS) to a total volume of 30–35 ml. It was then carefully layered into 20 ml of Ficoll Histopaque 1077 (Sigma-Aldrich, USA) in a 50 ml conical tube. Subsequently, the tube was put into a centrifuge at 300xg for 30 minutes. The PBMC layer was obtained in the form of a buffy coat layer. Using a sterile plastic pipette, the PBMCs were carefully taken and put into another 50 ml conical tube. Furthermore, PBMC was added with PBS + 2% FBS in a ratio of 1: 1, then stirred until homogeneous and centrifuged at 300xg for 7 minutes. This step was repeated with the supernatant removed, 15 ml of PBS + 2% FBS was added to the precipitate formed at the bottom of the tube and centrifuged at 300xg for 7 minutes. Finally, the supernatant was removed, and the sediment was dissolved with a basal medium. Cells were concentrated up to 5×10 ^6^ cells/ml.

### Isolation and culture of endothelial progenitor cells

To separate EPCs from PBMCs, we used standard protocols ^18^. Briefly, PBMCs isolated from blood samples in a concentration of 5×10 ^6^ cells/mL were collected in Stemline II Hematopoietic Stem Cell Expansion Medium (Sigma-Aldrich, USA) supplemented with endothelial basal medium (EBM) containing 40 ng/ml of vascular endothelial growth factor (VEGF) and 15% FBS. Then PBMCs were seeded in the six-well plate with fibronectin coating. The cultures were maintained with a humidified atmosphere at 37°C and 5% carbon dioxide. Forty-eight hours after seeding, we separated the medium liquid containing the non-adherent cells from the adherent cells attached to the bottom of the plate. All the medium liquid containing the non-adherent cells was collected into one tube, centrifuged with a spin at 300xg for 7 minutes, and the supernatant was discarded. The precipitate formed was dissolved with basal medium and supplement with a concentration of 1×10 ^6 ^cells/ml. We confirmed the cells as EPCs through immunofluorescence tests using fluorescein isothiocyanate (FITC) mouse anti-human CD34 monoclonal antibody (cat. no. 343604; Biolegend, USA).

### Treatment groups

Isolated EPCs were divided into three treatment groups and one negative control group. The treatment groups were divided into 1) captopril 1 μM, 10 μM, and 100 μM; 2) lisinopril 1 μM, 10 μM, and 100 μM; and 3) ramipril 1 μM, 10 μM, and 100 μM. The cultures were maintained with a humidified atmosphere at 37°C and 5% carbon dioxide for 48 hours. Each data point represented the mean value of quadruplicate cultures.

### Migration assay

We used the Boyden chamber migration assay to measure ECP migration. Briefly, migration chambers with 8μm pore-size filters were placed in 24-well plates. Using 1 mmol/L EDTA in PBS, isolated EPCs were detached and then centrifuged at 400xg for ten minutes. EPCs were seeded in the upper chamber (5×10 ^5^/ml in serum-free medium), and the lower compartment of the Boyden chamber was filled with endothelial basal medium. After 24 hours of incubation at 37°C, we scraped off non-migratory cells on the upper chamber with cotton swabs. The migration chamber was put into a new basal medium and added with 500μL of trypsin + EDTA 0.5% solution. After 10 minutes of incubation, we verified using a light microscope to ensure more than 90% of adhering cells were released from the lower surface of the migration chambers. For quantification, the cells were harvested and stained with trypan blue/Giemsa. Migrated ECPs were counted using an TC20 automated cell counter (Bio-Rad, USA).

### Statistical analyses

Continuous data were presented as mean ± SD. Multiple experimental group analysis of total migrated EPCs was performed using analysis of variance (ANOVA). A p-value of less than 0.05 was considered statistically significant. All statistical analysis was completed using SPSS version 25.0 for Windows.

## Results

### CD34 expression and migration capability of endothelial progenitor cells

**Figure 1.**
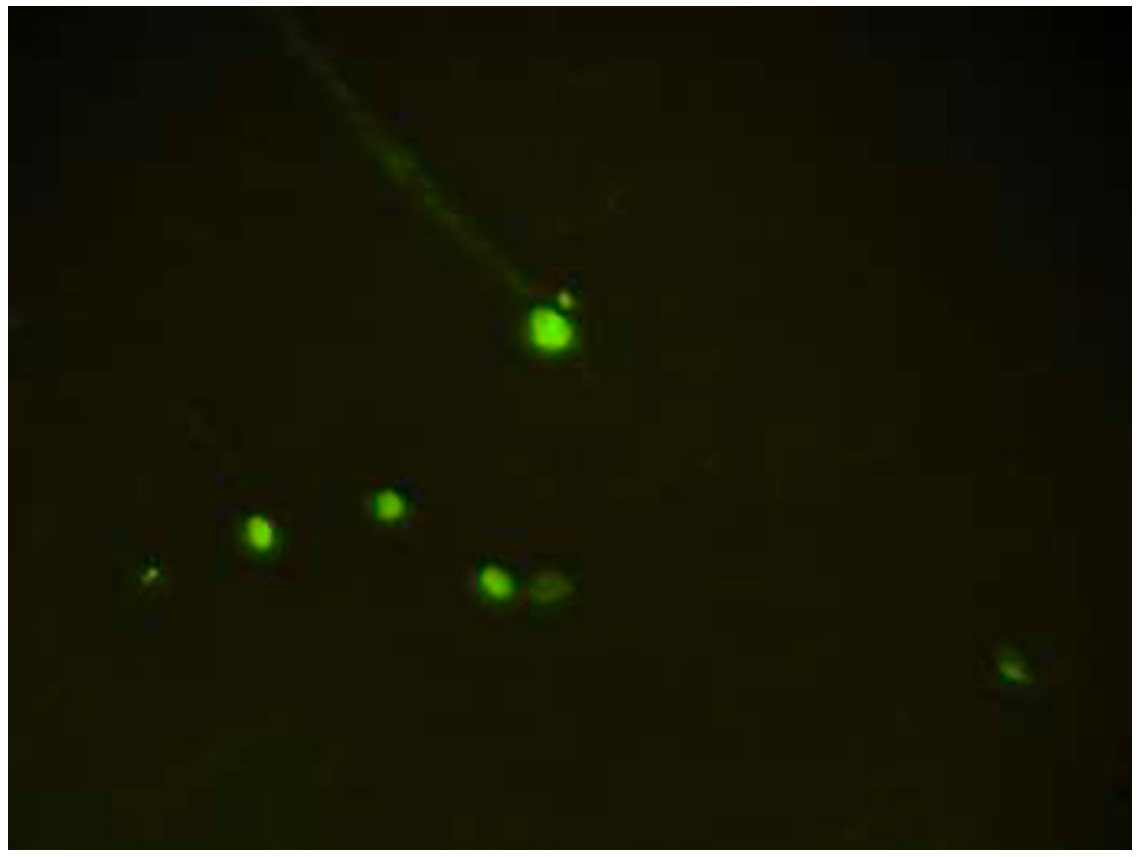
CD34 expression was characterized by the presence of green luminescence in endothelial progenitor cell culture.

**Figure 2.**
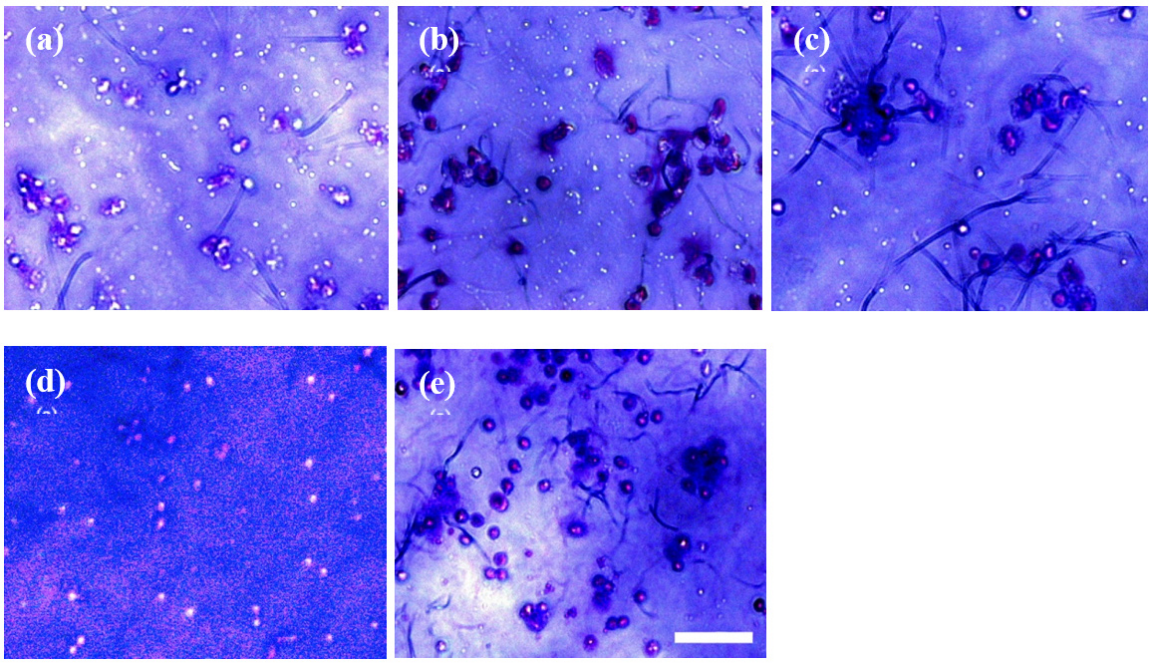
Light-inverted microscope view of endothelial progenitor cells under 48 h-treatment of ( **a**) 100 mM captopril, ( **b**) 100 mM lisinopril, ( **c**) 100 mM ramipril, ( **d**) negative control (medium only), and ( **e**) positive control (100 ng/mL VEGF). White bar represents 100µM.

CD34 is a positive marker for EPCs, and CD34 expression was found in the early to mature culture of EPCs. CD34 expression was characterized by the presence of green luminescence using a fluorescence microscope, indicating the presence of EPCs, as shown in Figure 1 ^19^. The migration capability of EPCs was evaluated by calculating the number of cells that moved from the upper chamber to the membrane facing the lower chamber with Giemsa staining ( Figure 2).

### ACE inhibitors increased endothelial progenitor cells migration

**Figure 3.**
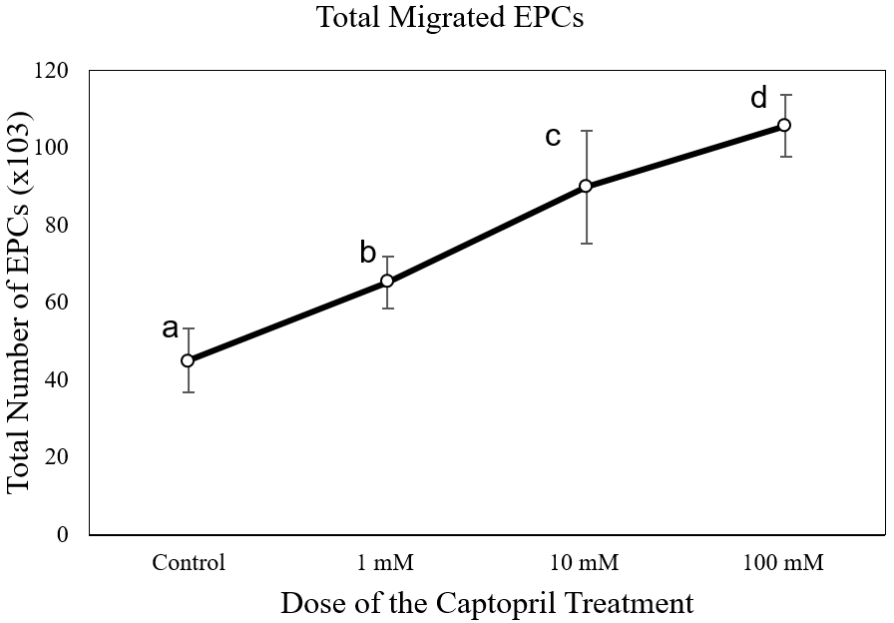
Total migrated endothelial progenitor cells (EPCs) on increasing dose of captopril treatment. Total migrated cells are expressed as mean ± SD (n = 4). Different annotations ^(a,b,c,d)^ denounce significant difference in ANOVA test (p<0.05).

**Figure 4.**
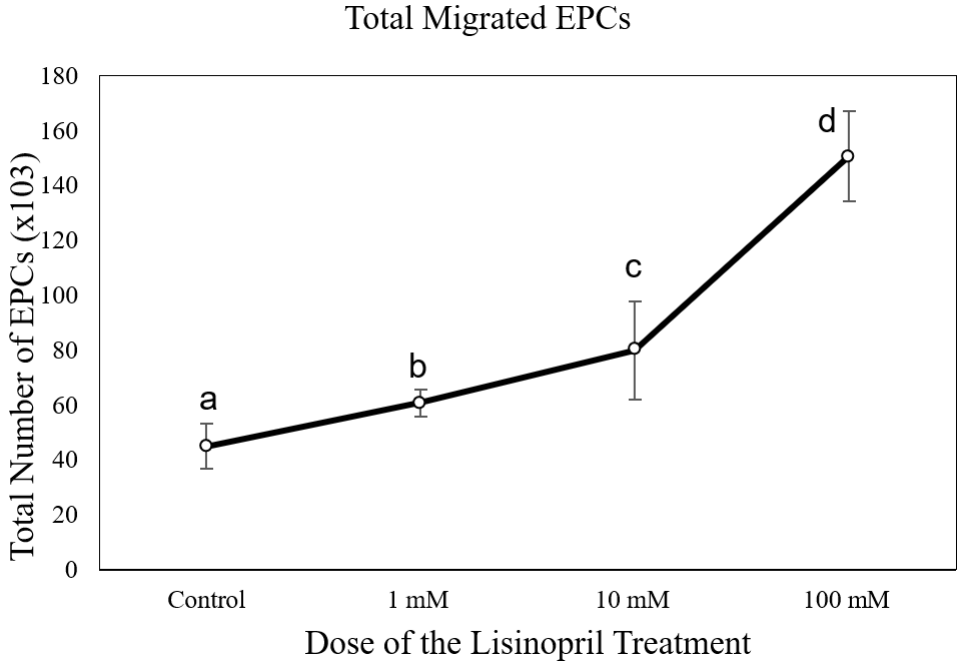
Total migrated endothelial progenitor cells (EPCs) on increasing dose of lisinopril treatment. Total migrated cells are expressed as mean ± SD (n = 4). Different annotations ^(a,b,c,d)^ denounce significant difference in ANOVA test (p<0.05).

**Figure 5.**
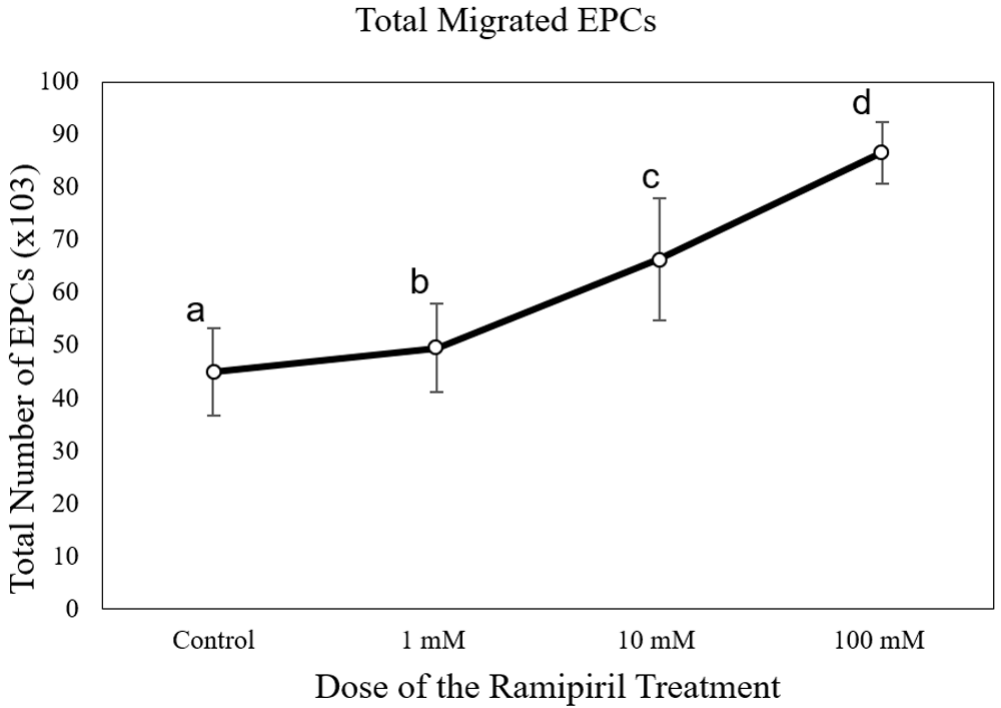
Total migrated endothelial progenitor cells (EPCs) on increasing dose of ramipiril treatment. Total migrated cells are expressed as mean ± SD (n = 4). Different annotations ^(a,b,c,d) ^denounce significant difference in ANOVA test (p<0.05).

The number of EPC migrations in the captopril-treated group at different doses (65,250 ± 6,750 cells at 1 mM; 90,000 ± 16,837 cells at 10mM; and 105,750 ± 8112 cells at 100 mM) was significantly higher than the control group (43,714 ± 7,216 cells) (p < 0.05) ( Figure 3). The number of EPC migrations in the lisinopril-treated group at different doses (60,750 ± 5,030 cells at 1 mM; 79,071 ± 2,043 cells at 10mM; and 150,750 ± 16,380 cells at 100 mM) was significantly higher than the control group (43,714 ± 7,216 cells) (p < 0.05) ( Figure 4). The number of EPC migrations in the ramipril-treated group at different doses (49,500 ± 8,400 cells at 1 mM; 64,285 ± 11,824 cells at 10mM; and 86,625 ± 5,845 cells at 100 mM) was significantly higher than the control group (43,714 ± 7,216 cells) (p < 0.05) ( Figure 5).

**Figure 6.**
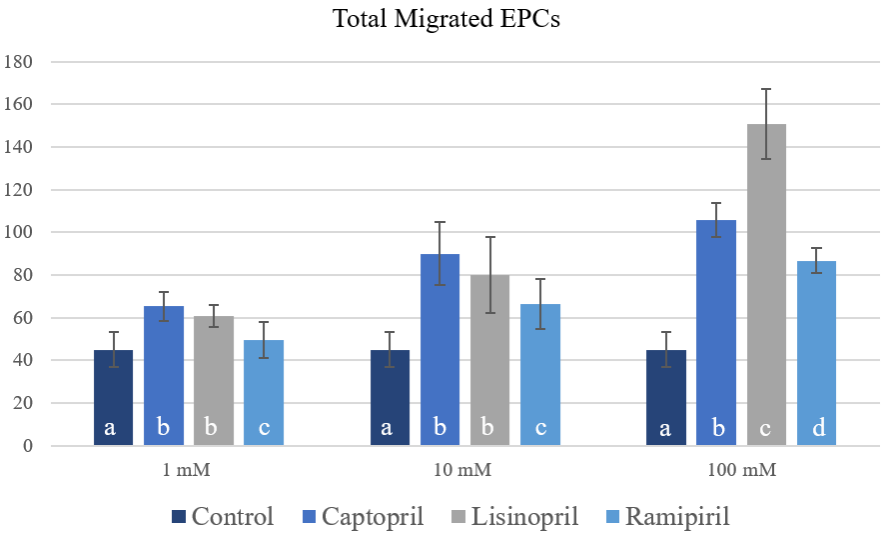
Total migrated endothelial progenitor cells (ECPs) on increasing dose of captopril, lisinopril and ramipiril. Total migrated cells are expressed as mean ± SD (n = 4). Different annotations ^(a,b,c,d)^ denounce significant difference in ANOVA test (p<0.05).

The increase in the migration of EPCs was consistent with the increase in the dose of ACE inhibitor. Captopril at doses of 1 mM and 10 mM had a higher migration effect than lisinopril and ramipril at the same doses (p < 0.05). Meanwhile, lisinopril at a dose of 100mM had the highest migration effect (p < 0.05) ( Figure 6).

## Discussion

In this present study, we demonstrated that captopril, lisinopril, and ramipril therapy in EPC cultures from CAD patients was associated with improved migration of EPCs. This study showed that ACE inhibitor treatment increases EPCs migration in a dose-dependent manner. At the doses of 1 mM and 10 mM, there was no significant difference in EPCs migration between captopril and lisinopril. However, both of them exceeded the results of ramipril at the same dose. Meanwhile, lisinopril at the dose of 100 mM had a superior outcome compared to captopril and ramipril at the same dose.

Circulating EPCs are derived from hematopoietic stem cells produced in the bone marrow, which can repair endothelial dysfunction through endogenous mechanisms. In patients with CAD, the number and migration capacity of ECPs are decreased, and thus they are unable to maintain adequate endothelial stability ^6, 20– 22^. During ischemic conditions, EPCs are known to play an essential role in reendothelization and neovascularization. Animal and clinical studies have shown that EPCs contribute up to 25% of newly formed vascular endothelial cells after ischemic conditions ^23, 24^.

Several pharmacological agents have reported the beneficial effects on EPCs, such as HMG-CoA reductase inhibitors/statin ^25, 26^, one of which has been demonstrated by our previous study ^16^, peroxisome proliferator-activated receptor (PPAR) agonists ^27^, dihydropyridine calcium channel blocker ^28^, and angiotensin II receptor antagonists (ARB) ^29^. Antioxidative agents with anti-inflammatory properties, such as ginsenoside, salvianolic acids, berberine, Ginkgo biloba, resveratrol, and puerarin, also have been found to increase the number or functional activity of EPCs ^30^. ACE inhibitors, which are widely used in cardiovascular therapy, such as for hypertension and congestive heart failure, may have a potential role in restoring the role of EPCs in repair, healing, and neovascularization ^11, 31^. Several studies have demonstrated the role of ACE inhibitors in increasing the number and function of EPCs in patients with hypertension and stable CAD ^13, 15^. Each of the ACE inhibitors has a different chemical functional group, which may explain the varying effects of different ACE inhibitor types in several studies, either *in vitro* or *in vivo*. Sulfhydryl-containing ACE inhibitors are known to be the most effective compared to other types of ACE inhibitors ^31– 34^. Captopril has one sulfhydryl group, and zofenopril has two sulfhydryl groups to coordinate the zinc ion of the active side, whereas lisinopril, ramipril, and enalapril do not have sulfhydryl groups ^35– 38^. Sulfhydryl-containing ACE inhibitors can reduce oxidative stress and stimulate nitric oxide (NO) activity in human endothelial cells ^39^ and patients with primary hypertension ^40^. *In vitro* studies have shown that zofenopril is more effective compared to enalapril in preventing foam cell formation and thereby slowing atherosclerosis. In addition, zofenopril can also reduce reactive oxygen species and increase NO production in the endothelium ^37, 41– 44^

The finding that ACE inhibition therapy augmented the number of circulating EPCs in patients with CAD, and also enhanced EPCs functional activity, may provide a novel strategy to improve neovascularization and reendothelialization after ischemia, thereby providing a therapeutic concept to improve EPC numbers and functions in patients with CAD.

## Conclusion

Captopril, ramipril, and lisinopril were shown to increase EPC migration in a dose-dependent manner. Low-dose (1 mM) and medium-dose (10 mM) captopril had a larger effect on ECP migration than lisinopril and ramipril. Meanwhile, high-dose lisinopril (100mM) had the highest migration effect, suggesting it may be preferable for promoting EPC migration in CAD patients.

## Data availability

### Underlying data

Figshare: Dataset for Enhancement of EPC migration by high-dose lisinopril is superior compared to captopril and ramipril. https://doi.org/10.6084/m9.figshare.13130303.v2 ^19^

This project contains the following underlying data:

- Transwell_Migration_Assay_Dataset.xlsx
- Image Repository.zip (original, unedited microscopy images in JPG format)
- Clinical and demographic data of study population.docx

Data are available under the terms of the Creative Commons Attribution 4.0 International license (CC-BY 4.0).
