# Supplementary material for: Enhancement of EPC migration by high-dose lisinopril is superior compared to captopril and ramipril
Source: F1000Res. 2021 Jan 11;10:15. [Version 1] doi: 10.12688/f1000research.26395.1 (PMC8515492; doi:10.12688/f1000research.26395.1)
Supplement: Peer review history

# Peer review: Reviewer response for version 1

**DOI:** 10.5256/f1000research.29142.r96122

**Published:** 2021-10-13

**Authors:** Dyana Sarvasti

**Affiliations:** 1 Department of Internal Medicine, Faculty of Medicine, Widya Mandala Catholic University, Surabaya, Indonesia

This study explores the role of ACE inhibitors (captopril, lisinopril, and ramipril) in improving the migratory capabilities of EPCs in coronary artery disease (CAD). Overall, the research published by this author is very interesting. However, several things need to be added to this research report so that readers can understand it better. For example, in the abstract, it is necessary to add the characteristics of the patients included in this study so that the reader can understand the study's outline.   

 In the research method, it is also necessary to explain whether or not the patients included in this study are already on ACE inhibitor therapy. Was the number of patients included in the study (eight patients) sufficient to conclude? What about the effect of other therapies (not ACE inhibitors) that the patient had taken before being included in the study? How to “clean up” the various confounders in the study? Some of these questions may need to be added and explained in this research report.

 In addition, there are some inconsistencies in the writing of abbreviations and mistypes that need to be corrected. Here are some points related to this:

- “ *However, the efficacies of different ACE inhibitors in improving the migratory capabilities of ECPs in coronary artery disease (CAD) patients **is **unclear*.” The singular verb “ **is”** does not appear to agree with the plural subject **"patients" **or **"the efficacies"**.
- *“Keywords: ACE Inhibitors, **C**oronary artery disease, **E**ndothelial progenitor cells, **M**igration*.” It should be in lowercase.
- The description of the **abbreviation CAD** differs between the description in the abstract and the introduction.
- The author is inconsistent in writing the abbreviation for endothelial progenitor cells. Sometimes it says “ **EPC”**, but in other parts, it says “ **ECP”**.
- “ *It is suggested that intracellular damage and impaired redox balance in EPCs due to oxidative stress **are the predisposes of **imbalance in vascular pathology*.” The phrase “ **are the predisposes of”** maybe wordy. Consider changing to “ **predisposes to**”.
- “ *Forty-eight hours after seeding, we separated the medium liquid **ontaining** the non-adherent cells from……*.”. It should be “ **containing**”.
- “ *The precipitate formed was dissolved with basal medium and **supplement **with*……”. It should be “ **supplemented**”.
- The author is inconsistent in writing the unit dose of the drug. In the abstract, it says “ **mM”**, but in other parts, it says “ **μM”**.
- “ *Migrated EPCs were counted using **an** TC20 automated cell counter (Bio-Rad, USA)*”. It should be “ **a**”.

Is the work clearly and accurately presented and does it cite the current literature?

Yes

If applicable, is the statistical analysis and its interpretation appropriate?

I cannot comment. A qualified statistician is required.

Are all the source data underlying the results available to ensure full reproducibility?

Yes

Is the study design appropriate and is the work technically sound?

Yes

Are the conclusions drawn adequately supported by the results?

Yes

Are sufficient details of methods and analysis provided to allow replication by others?

Yes

Reviewer Expertise:

Cardiovascular prevention and rehabilitation.

I confirm that I have read this submission and believe that I have an appropriate level of expertise to confirm that it is of an acceptable scientific standard.

# Peer review: Reviewer response for version 1

**DOI:** 10.5256/f1000research.29142.r84488

**Published:** 2021-05-17

**Authors:** Mehdi Hassanpour

**Affiliations:** 1 Department of Biochemistry and Clinical Laboratories, Tabriz University of Medical Sciences, Tabriz, Iran

The manuscript entitled “ ***Enhancement of EPC migration by high-dose lisinopril is superior compared to captopril and ramipril***” seems an interesting topic in the field of cardiac and vessel cell biology. However, there are some points that need revision before indexing.

**Reviewer comments:**

1. The topic is interesting and the aim of the study is commendable, but this article has some typos errors, abbreviations, and grammatical issues and needs editing.
2. The ***EPC*** should be in complete form in the title of the article.
3. As a result of EPC confirmation through immunoﬂuorescence tests, what percentage of cultured cells were CD34 positive?
4. The underlying molecular mechanism and involved signaling pathways of Lisinopril on EPC migration were neglected and should be discussed.

Is the work clearly and accurately presented and does it cite the current literature?

Yes

If applicable, is the statistical analysis and its interpretation appropriate?

Yes

Are all the source data underlying the results available to ensure full reproducibility?

Yes

Is the study design appropriate and is the work technically sound?

Yes

Are the conclusions drawn adequately supported by the results?

Yes

Are sufficient details of methods and analysis provided to allow replication by others?

Yes

Reviewer Expertise:

Clinical Biochemistry

I confirm that I have read this submission and believe that I have an appropriate level of expertise to confirm that it is of an acceptable scientific standard.
